# Measurement of food consumption growth in Vietnamese households: The role of classifying predicted and transitory income shocks by asset accumulation

**DOI:** 10.1371/journal.pone.0333596

**Published:** 2025-10-07

**Authors:** Ka Son La, Ha Duc Vu

**Affiliations:** 1 School of Economics and Management, Southeast University, Nanjing, China; 2 Thai Nguyen University of Economics and Business Administration, Thai Nguyen, Vietnam; Aalto University, FINLAND

## Abstract

The study measures the food consumption growth in Vietnamese households by classifying income and assets or consumption habits. The FE, RE, and Sys-GMM methods estimate dynamic data from the VARHS (2008–2018) survey of 1,915 households in 466 communes. The results show heterogeneity in marginal propensities to food consumption growth (MPCs) by asset level and by breaking down income changes (current income, predictable income and transitory income shocks). The MPCs, in response to transitory income shocks, are higher for poorer households than for richer ones. Additionally, classification by growth rates of assets reveals new characteristics in household food consumption growth. For household groups with slower growth rates of assets, the MPCs decrease, and other factors such as asset growth, ethnicity, and household size become more important in explaining food consumption growth, especially for predictable income shocks. Ultimately, food consumption habits do not explain changes in household food consumption growth.

## 1. Introduction

The findings revealed that income and asset in changes are important determinants of the food consumption behavior of Vietnamese households. Food consumption is explained in various ways, especially the role of income shocks. Nguyễn, White, and Ma (2019) [1] noted the covariate income shocks. On the other hand, [2] shows that permanent income shocks are highly significant. Specifically, income shocks have a strong impact on households’ food security consumption during the COVID-19 pandemic [3]. In details, [4] show that marginal propensity to food consume (MPCs) is larger for positive than negative income shocks during COVID-19. Beside income, the role of assets has been emphasized as a buffer against income shock to smoothen food consumption [Nguyễn, White & Ma, 2019]. Accumulation of finance and assets mitigated economic volatility for households [5]. Asset growth and poverty reduction are significant in Vietnamese households [6,7]. In particular, these studies do not reflect how the asset differentiation related to MPCs for Vietnamese households.

To explain consumption growth more, [8] with microdata indicate that consumption growth affected by changes in income and assets, dismissing consumption habits. The studies’ MPC shows that consumption is highly sensitive to current income changes [9,10], anticipated shocks [11,12], and temporary shocks [13,14]. The MPC with classification shows that MPC is higher in poorer groups [15,16]. Arellano et al. (2024) [17] show a nonlinear relationship between consumption and income. Heterogeneity in consumption with MPC measurement by asset quintiles is quite common [18–20]. Classifying by wealth implies that wealth and income inequality have transformed into consumption inequality [21]. Many studies above have not considered the role of asset accumulation rates. This issue needs further analysis on the role of asset accumulation rate in changing asset ranking and its impact on expected consumption. In Vietnam, with a rapidly growing and structurally changing economy [22], it is necessary to consider the role of the growth rate of assets in classifying MPC rather than only by wealth. Building on these insights from the general consumption literature, our study extends the analysis to food consumption growth in Vietnamese households, where asset accumulation plays a particularly important role.

First, this study measures food consumption growth by different income changes and classified assets in Vietnamese households. The marginal propensity to consumption growth (MPCs) is estimated with an extended Euler equation. By following this approach, detailed income in changes are measured from three perspectives: current, anticipated shocks, and transitory shock. These models give a more complete analysis than some previous studies in Vietnam.

Second, the classification of MPCs by growth rates of assets is estimated, besides measuring MPCs by asset index [15,18]. Using dynamic data from the Vietnam Access to Resources Household Survey (VARHS) from 2008 to 2018 allows for better classification of households by growth rate of assets. Moreover, the dynamic data VARHS, regression by the system-GMM method, allows for testing the hypothesis that consumption habits do not significantly influence consumption growth with microdata in Vietnamese households [8,23].

The remainder of the study is organized into several sections. Section 2 provides an overview of the theory of marginal propensity to consumption growth (MPCs) and its relationship with wealth and consumption growth. Section 3 focuses on households in Vietnam. Section 4 introduces the model to integrate MPCs with asset indices and food consumption growth estimation. Section 5 discusses the results of MPCs, asset indices, as well as food consumption growth in Vietnamese households. Finally, Section 6 concludes and policy implications.

## 2. Literature review

### Measurement of consumption growth

According to the PIH/LCIH hypothesis, household consumption is based on expectations of permanent income to smooth consumption. The estimation model for consumption growth shows that changes are a random process, meaning consumption growth cannot be predicted [24]. In later studies, consumption growth can be measured and explained by consumption habits and current income. Liquidity constraints are reasonable households that rely on current income for consumption [25,26]. Another explanation suggests that consumption is excessive sensitive to income according to the ‘rule of thumb’ [27,28]. Havranek et al. (2020) [29] studied a meta-analysis on the excessive sensitivity to income to explain the liquidity constraints that force households to rely on current income instead of the ‘rule of thumb’. The role of excessive sensitivity needs to be analyzed through various states of income changes to measure the impact on consumption growth. While these theories address consumption in general, they also provide the foundation for examining food consumption growth, which is the focus of this study.

Besides excessive sensitivity, consumption growth can be explained by consumption habits (lag consumption growth), as shown by [30], although [31] did not find such a link for U.S. households. Basu and Kimball (2002) [32] show that consumption growth has habits of past consumption. Sommer (2007) [33] demonstrates that consumption habits are present in macroeconomic data from OECD countries. Kiley (2010) [34] finds that consumer habits and the “rule of thumb” are significant. Using macroeconomic data, Everaert and Pozzi (2014) [35] tested the hypothesis regarding the predictability of consumption growth for OECD countries and generated reliable results. Havranek et al. (2017) [23], by a meta-analysis, show that estimates of consumption growth due to habit formation are biased, and the reasonable cause is imperfect information. Carroll et al. (2020) [8] explain consumption growth as driven by expectations of assets and income rather than habits, by household data. This hypothesis is tested again in the case of Vietnamese households.

### Heterogeneity MPCs and asset classification

The marginal propensity to consumption growth (MPCs) responds depending on the status of income changes, whether their response are temporary shocks or anticipated income changes. MPC reacts more strongly to temporary income shocks than long-term income changes [36–38]. Similarly, transitory income shocks are substantial fluctuations in consumption growth for financially vulnerable groups [9–11,13,14]. In detail, unexpected temporary income changes are excessive sensitivity [39]. Anticipated income shocks, especially during COVID-19, positive shocks had more affection than negative [4,12]. Moreover, MPCs can increase more by economic expectations than direct income changes [40]. Although these analyses have detailed the different types of income changes and excessive sensitivity, further study is needed to detail MPCs in relation to asset differentiation. Although these analyses focus on consumption in general, such mechanisms are expected to be even more relevant for food consumption, particularly in low- and middle-income households where food expenditure dominates.

Wealth is closely linked to consumption when MPCs respond to classification by wealth. Wealth changes are sensitive to consumption, particularly for households with cash in hand [15,41]. MPCs vary with wealth quintiles such as groups with fewer assets tend to have higher MPCs because of income shocks [18], with wealthier households having lower MPCs [42,43]. Hand-to-mouth households quickly change their consumption when income changes, while those with more illiquid assets, like houses, respond more slowly [19]. The issue of asset and income inequality clearly leads to consumption inequality [21]. Additionally, heterogeneity in consumption is affected by changes in socioeconomics and psychological factors in these differences in MPCs [16,44,45]. Although MPC has studied cases related to asset classification, research has not fully explained how asset accumulation affects consumption.

Consumption behavior can be described in some ways by the difference between asset index and growth rate asset in Vietnam. Common asset accumulation reduced consumption risks through asset ownership and the lessened effects of economic shocks that asset stability provides [5,46]. According to [6], asset growth is helpful in reducing poverty in rural areas. Women and education can assist households to build up assets and raise income [47] and asset capabilities [7]. These studies do not investigate the effect of asset accumulation on MPCs. Our study fills this gap by specifically linking asset accumulation to food consumption growth.

It is interesting to measure MPCs based on asset indices and the growth rate of asset classification in developing countries like Vietnam. But one of its biggest challenges is a shortage of collected data for developing countries [48,49]. Except for [1], who relied on VARHS, most studies have used cross-sectional data (Vietnam Household Living Standards Survey) that restricts the measure of consumption growth. Asset indices are inspirations used in developing countries such as Vietnam instead of wealth indices in developed countries. Filmer (2012) [50] reports that both types of indices present similar results. According to [51], asset indices are sometimes inaccurate, but asset indices can help clearly reduce poverty [49,52]. The VARHS is valuable for dynamic analyses because it allows the calculation of the difference in the growth rate of assets and asset index over a period.

Overview, the research focuses on several gaps in measuring household food consumption growth in Vietnam. First, how is food consumption growth impacted by different types of MPCs (including short-term to long-term, positive and negative)? Next, how do the types of MPCs differ among household food consumption groups by asset index? Then, how does the role of asset accumulation respond to food consumption, or how does the rate of asset accumulation affect the asset index? Finally, MPCs clarified by growth rate of asset accumulation will provide a more detailed view of household food consumption responses to structural economic changes in developing countries. Lately, food consumption habits should test at the household level in Vietnam.

## 3. Research methodology

### 3.1. The role of assets in marginal propensity to food consumption growth

The MPCs are estimated by the change in log food consumption in response to a change in log income. In this study, all equations are applied to food consumption growth rather than general consumption growth, to reflect the specific focus on Vietnamese households. Extended Euler equations have added some factors, such as the asset index and demographic characteristics. Food consumption changes are explained in more detail. Studies by [9] and [18] demonstrate that consumption responses with different types ofincome changes. The first step is to estimate the basic Euler equation:


$$\Delta ln(C_{i,t})=\alpha +\beta_{\text{1}}\Delta ln(Y_{i,t})+{\delta A}_{i,t}+{\gamma Z}_{i,t}+v_{i,t}$$ (1)

The variables of consumption (*C*_*i,t*_), income (*Y*_*i,t*_), and asset index (*A*_*i,t*_) in changes are measured between periods *t* and *t* − 2. The vector *Z*_*i,t*_ includes demographic controls, such as the age of the household head, gender, household size, number of laborers, race, region, and locality.

From equation (1) it is possible to estimate the impact of the current income changes. How evaluate the impact of the MPCs from predictable income changes [12,18] and MPCs from transitory income shocks [11,13,14]? It is necessary to analyze the income process for income changes by lagging income changes and including various demographic controls:


$$\Delta ln(Y_{i,t})=\phi +\rho \Delta ln(Y_{i,t}-\text{2})+{\zeta Z}_{i,t}+\epsilon_{i,t}$$ (2)

In equation 2, predictable income is measured using past income shocks to instrument future income shocks. The predicted income (Δ*Y^*_*i,t*_) value is *E*_*t*_ Δln(*Y*_*i,t−2*_), and residuals represent transitory income shocks (*∊*_*i,t*_). The extended Euler equations (1) combined (2) can be divided into two components: predictable and transitory shocks. These equations are outlined below:


$$\Delta ln(C_{i,t})=\alpha +\beta \Delta ln({\hat{Y}}_{i,t})\;+\;{\delta A}_{i,t}+{\gamma Z}_{i,t}+v_{i,t}$$ (3)

$$\Delta ln(C_{i,t})=\alpha +\beta \Delta ln(\epsilon_{i,t})+\;{\delta A}_{i,t}+{\gamma Z}_{i,t}+\upsilon_{i,t}$$ (4)

The extended Euler equation cannot fully address the issue of heterogeneity in the income changes response to consumption. Wealth inequality is correlated with income inequality and consumption inequality [15,19,21]. The heterogeneity of assets and income is estimated by analyzing income changes relative to asset index quintiles. Equations (1), (3), and (4) with asset index quintiles ($A_{i,t}^{Q_{j,t}}$) are rewritten as follows:


$$\Delta \text{ln}\left(C_{i,t}\right)=\alpha +\beta_{j}\left(\Delta \text{ln}\left(Y_{i,t}\right)\times A_{iX}Q_{j,t}\right)+{\delta A}_{i,t}\;+\;{\gamma Z}_{i,t}+v_{i,t}$$ (5)

$$\Delta \text{ln}\left(C_{i,t}\right)=\alpha +\beta_{j}\left(\Delta \text{ln}\left({\hat{Y}}_{i,t}\right)\times A_{iX}Q_{j,t}\right)+{\delta A}_{i,t}\;+\;{\gamma Z}_{i,t}+v_{i,t}$$ (6)

$$\Delta \text{ln}\left(C_{i,t}\right)=\alpha +\beta_{j}\left(\Delta \text{ln}\left(\epsilon_{i,t}\right)\times A_{iX}Q_{j,t}\right)+{\delta A}_{i,t}\;+\;{\gamma Z}_{i,t}+v_{i,t}$$ (7)

In addition, how does the growth rate of the asset index (*AG*_*i,t*_) correlate with the MPCs, especially in rapidly developing countries like Vietnam? Income change is classified based on quintiles of the asset index growth rates. Equations (1), (3), and (4) are updated to incorporate these growth rate quintiles (${AG}_{i,t}^{Q_{j,t}}$) and are rewritten as follows:


$$\Delta ln(C_{i,t})=\alpha +\beta_{j}(\Delta ln(Y_{i,t})\times {AG}_{i,t}\times Q_{j,t})+{\delta AG}_{i,t}+{\gamma Z}_{i,t}+v_{i,t}$$ (8)

$$\Delta ln(C_{i,t})=\alpha +\beta_{j}(\Delta ln({\hat{Y}}_{i,t})\times {AG}_{i,t}\times Q_{j,t})+{\delta AG}_{i,t}+{\gamma Z}_{i,t}+v_{i,t}$$ (9)

$$\Delta ln(C_{i,t})=\alpha +\beta_{j}(\Delta ln(\epsilon_{i,t})\times {AG}_{i,t}\times Q_{j,t})+{\delta AG}_{i,t}+{\gamma Z}_{i,t}+v_{i,t}$$ (10)

Equations (3) to (8) provide a detailed explanation of the heterogeneity in food consumption growth among Vietnamese households concerning MPCs and asset classification. These equations focus on classification by asset and the rate of asset growth in influencing food consumption growth.

### 3.2. Explanation of food consumption growth

MPCs and wealth explain consumption growth, but studies at the household level show that consumption habits have little impact. [8] find that although there is a small, significant lag in consumption, this is mainly because households fail to update information rather than due to consumption habits. To estimate the model for Vietnamese household food consumption growth more accurately, this study combines the consumption growth model from [8] with asset classification. Note that in Equation (1), both the asset index and asset accumulation are estimated together. In the equation below, asset categories are estimated separately to provide detail and avoid endogeneity.


$$\Delta ln(C_{i,t})=\alpha +\chi (\Delta ln\left(C_{i,t-\text{2}}\right)+\beta_{\text{1}}\Delta \text{ln}(Y_{i,t})+{\delta A}_{i,t}+{\gamma Z}_{i,t}+v_{i,t}$$ (11)

$$\Delta ln(C_{i,t})=\alpha +\chi (\Delta ln\left(C_{i,t-\text{2}}\right)+\beta_{\text{1}}\Delta \text{ln}(Y_{i,t})+{\delta AG}_{i,t}+{\gamma Z}_{i,t}+v_{i,t}$$ (12)

Equations (11) and (12) are estimated by dynamic panel data so they face estimation challenges due to autocorrelation [17]. Most studies on consumption use cross-sectional data in Vietnam so that the problem of autocorrelation is not considered [3,53]. The different types of data lead to a unique model. This makes it important to choose the right estimation methods. Dynan (2000) [31] addressed the endogeneity issue in microdata and solved it by the GMM method. In dynamic panel data, the endogeneity problem requires using instrumental variables, such as lagged values of consumption and income or combinations of the two, to resolve it [8].

## 4. Data of Vietnamese households

### 4.1. Descriptive of households

The Vietnam Access to Resources Household Survey (VARHS [https://www.wider.unu.edu/database/survey-data-growth-structural-transformation-and-rural-change-viet-nam-book, accessed on April 12, 2023]) was a source for the indicators of food consumption, income, asset index, and demographic factors in Vietnamese households. Designed by the United Nations University World Institute for Development Economics Research (UNU-WIDER) [54], VARHS data is a project in the Netherlands, that received support from the Central Institute for Economic Management (CIEM [https://ciem.org.vn/nghien-cuu-du-an/5964/vietnam-access-to-resources-household-survey-2012?newsgroup, accessed on April 12, 2023.] [55]) in Vietnam. In this study, we chose VARHS data instead of VHLSS because of the special repeat of the data. VHLSS is surveyed by the General Statistics Office every 2 years and is maintained regularly, with each nationwide survey covering 40,000 households, including detailed information on food consumption in over 9,000 households. However, households in VHLSS (9,000 households) are only repeated for about 20% of the next survey, so the number of households that are repeatedly observed is very small. In contrast, VARHS data is designed to continuously monitor over 10 years with 6 repeats. VARHS data is truly rare for developing countries like Vietnam. VARHS allows deep analysis for long-term growth indicators. The VARHS data includes more than 2,162 households from 466 communes across 12 provinces in Vietnam. Tarp (2017) [56] confirms that the VARHS statistical characteristics are equivalent to the Vietnam Household Living Standards Survey (VHLSS) data from the General Statistics Office of Vietnam. Data from ‘UNU-WIDER’, the study added the year 2018 and excluded the year 2006 to ensure that the households were consistent data.

The original data was obtained for 1,915 households who participated in six surveys from 2008–2018 with a total of 10,767 observations. The consumption variable is all food expenditure, and the income variable is the total real income from all sources. These valuable unit of currency is thousands of Vietnamese Dong (1000 VND). Household assets, then, serve as the basis for estimating the asset index. The mean estimates represent the average Vietnamese households over the period from 2008 to 2018, detailed in Table 1 below:

**Table 1:** Descriptive of Vietnamese households.

	2008	2010	2012	2014	2016	2018
Household Income	48288	85262	89487	115057	123030	181434
Food Expenditure	13691	15249	25507	26087	24505	31298
Asset index	0.120	0.300	0.290	0.300	0.240	0.080
Age	50.480	51.980	53.510	54.960	56.430	57.920
Education	1.240	1.300	1.350	1.430	1.350	1.300
Household Size	4.750	4.510	4.450	4.410	4.310	4.270
Labor Size	3.370	3.260	3.310	3.290	3.220	3.150

Note: Calculated from VARHS 2008–2018

Table 1 shows that income grew fast by three times (48288–181434) period 2008–2018 and food expenditure improved with more two times (13691–31298). The average age of head households who participated in the survey for the first time (50 years old) was young and is still in the workforce. The household education is low in primary and secondary school that may affect to ability to improve income. The household size was quite large due to the many households living in rural, but it decreased with the trend in labor. The statistical indicators of Vietnam’s households describe the quick change structure of the economic, similar to results in previous studies [57,58].

The average household asset index increased in 2008–2010, then decrease and the lowest value in 2018 which is lower than 2008 (0.08), but what contributed to the fluctuation of the average asset index? The average asset index was low and did not capture moves in household groups because some households have risen from the poor group to a better group or vice versa [5,46]. The average asset index may not reflect in detail the trend in food consumption and income growth when households rise out of poverty successfully [6]. Additionally, these assets might be stored in other forms hard to measure in rural households (cash, gold, or loans…). Thus, classification based on the rate of asset accumulation or other linking to food consumption and income is better idea than average asset index.

### 4.2. Classification of assets in marginal propensity to food consume growth

As mentioned in the literature review, household food consumption (*c*_*i,t*_) and income (*y*_*i,t*_) over time by asset groups show a close relationship. Variables of real food consumption (ln*C*_*i,t*_) and real income (ln*Y*_*i,t*_) form logarithmic to eliminate errors and are used for the next calculations. The perspective of growth is second order (Δln*C*_*i,t*_ = ln*C*_*i,t*_ - ln*C*_*i,t-2*_; Δln*Y*_*i,t*_ = ln*Y*_*i,t*_ - ln*Y*_*i,t-2*_). This research looks at how assets affect MPCs by classification. Normally, MPCs are grouped by wealth levels, but here the focus is on how asset growth rate impacts MPCs.

The quintile households by asset index show in wealth level (poor to rich). Poorer households are expected to react more to changes in food consumption. This way of grouping MPCs by asset index is common in developed countries [15,18]. Similarly, the asset index is a good way to differ MPCs in Vietnam. The groups by asset index are calculated by averaging over time to reduce changes in ranking.

The quintile households by the growth rate of the asset can create differences compared to tradition. Households can move ranks due to rapid asset accumulation over a long period (2008–2018). Households with fast asset accumulation may move from a lower to a higher asset index group. Do households at the same poverty or wealth level but with different asset accumulation rates have different MPCs? This classification emphasizes the expected food consumption of households regarding the accumulation rate. The group growth rate is calculated by averaging the differences between survey rounds.

In Table 2, the households in rich (quintiles 4 and 5) have high income and food consumption, and low income and food consumption in poor (quintiles 1, 2, and 3). Asset index quintiles show a clear trend income and food consumption growth over the 2008–2018 period. The difference in income between quintiles 3 & 4 has narrowed in 2018, possibly suggesting that the economic growth may be converging for these middling asset groups. Table 3 then explores the relationship between food consumption and income based on classification by asset accumulation rate.

**Table 2:** Mean of food consumption and income by asset index.

Cohorts		2008	2010	2012	2014	2016	2018
Quintile 1	*y*	28114	51273	54609	74169	78641	104954
	*c*	9585	11569	16978	19068	17210	23037
Quintile 2	*y*	43873	63381	79440	84853	100549	136435
	*c*	11170	13785	20991	22898	19438	27420
Quintile 3	*y*	46643	86888	94068	116498	117525	191847
	*c*	13400	13900	26205	26187	24049	31716
Quintile 4	*y*	55054	103352	104966	136653	140801	177099
	*c*	14826	16102	27951	27081	27486	34730
Quintile 5	*y*	68568	122627	115215	161714	175414	294713
	*c*	18234	19188	32188	32498	33943	39401

Note: Calculated from VARHS 2008–2018.

**Table 3:** Mean of food consumption and income by growth rate of assets.

Cohorts		2008	2010	2012	2014	2016	2018
Quintile 1	*y*	79164	124959	119464	161163	144612	218102
	*c*	19260	19027	29516	30804	26022	34244
Quintile 2	*y*	52905	87867	100741	122829	131773	196350
	*c*	13829	15067	27722	28631	27008	29485
Quintile 3	*y*	41530	71132	85124	105927	121539	167789
	*c*	12035	13977	25403	24999	25688	32983
Quintile 4	*y*	37016	70936	79190	89885	115225	154759
	*c*	11958	13616	22738	22189	24120	31297
Quintile 5	*y*	31069	69615	62011	95462	103070	173346
	*c*	9541	13206	21219	22401	19867	28657

Note: Calculated from VARHS 2008–2018.

**Fig 1:**
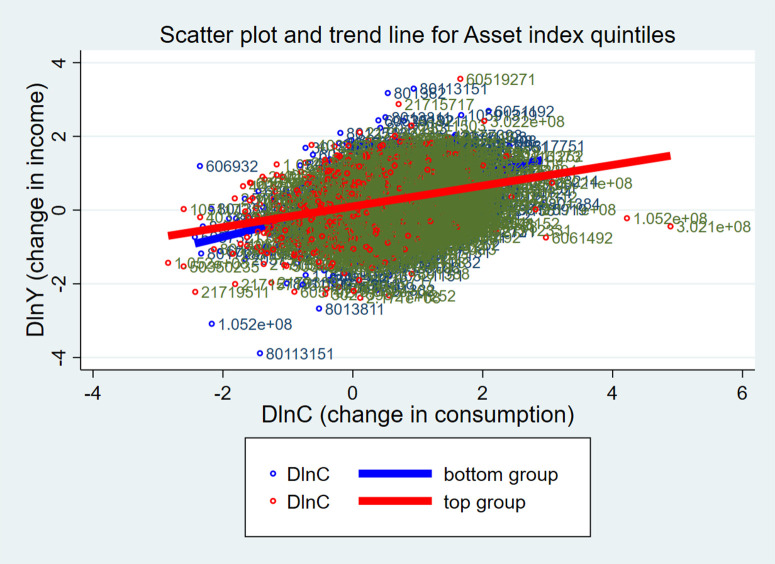
Food consumption and income growth by asset index.

**Fig 2:**
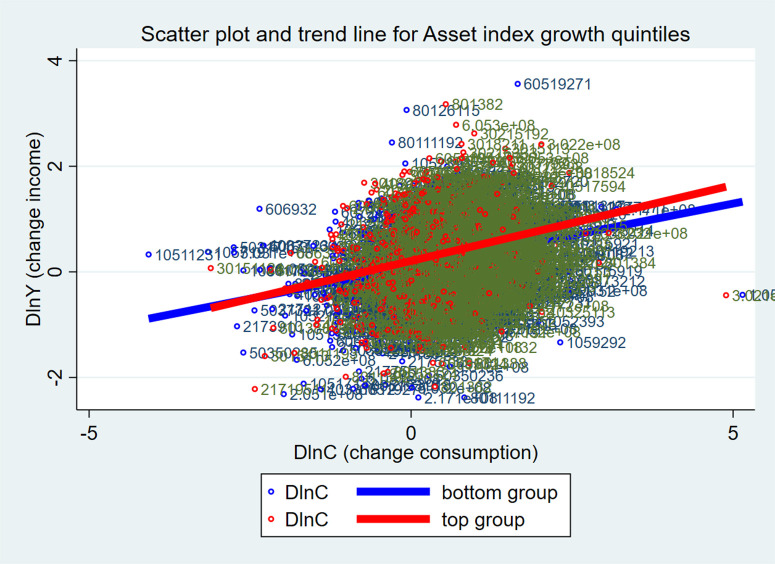
Food consumption and income growth by growth rate of assets.

According to the growth rate of assets in Table 3, results show that household lower income and food consumption levels are correlated with higher growth rates. That suggests households with lower income and food consumption are speeding up asset accumulation. Comparing Tables 2 and 3, households with higher asset indices have slower asset growth, while those with lower asset indices have faster growth. Grouping by asset index or asset accumulation rates shows that consumption and income maintain consistency in trends. Below is Fig 1, showing changes in food consumption and income (the highest and lowest groups) by asset index, and Fig 2, showing changes by asset accumulation rate.

The upward trend of the two graphs in Figs 1 and 2 means that income growth leads to food consumption growth. In Fig 1, comparing the wealthiest and poorest households in terms of assets, the poorest households (bottom group) are more represented with respect to change in income on food consumption growth. According to Fig 2, the group with the fastest rate of asset accumulation (top group) is more sensitive to income changes. Food consumption growth is subject to different kinds of effects from classification methods based on asset index and asset accumulation rate. The charts are really just observations; however, they are extremely intuitive, so estimation is needed to dive in and begin exploring the causes and effects as well as ensuring reliability.

## 5. Research results

### 5.1 Marginal propensity to food consumption growth with Euler equation

The coefficients are estimated by equation (1). Specifically, this equation investigates the role of different factors in determining how Vietnam’s household food consumption responds to economic shocks. Income growth, asset index growth, the age and gender of the household head, household size, number of laborers, and ethnicity are key factors. Other variables are considered such as region and locality. Equation (1) is estimated using dynamic panel data from VARHS, and two methods—Fixed Effects (FE) and Random Effects (RE)—are applied to address the issues of autocorrelation and heterogeneity in the data.

Table 4 shows some differences in results between columns 2 and 3 for the FE and RE methods, respectively. For example, the FE estimation excludes the “region” variable due to multi-collinearity by the “rural” variable. The asset index, number of laborers, and ethnicity variables show opposing significance between the FE and RE methods. The FE method is suitable for estimations because of the Hausman test value (0.016).

**Table 4:** Euler equation for food consumption growth.

Variable	FE	RE
	Coefficient	Coefficient
Δln*Y*	0.240***	0.275***
Asset Index	−0.077	−0.052***
ΔAsset Index	0.160***	0.159***
Year	0.005	−0.003
Rural	−0.006	0.020
Region	#	−0.004
Gender of head	−0.229	0.007
Ethnic	0.692**	0.056
Age of head	0.000	0.000
Married status	0.113	0.003
Education	0.037	0.019
Household size	0.095***	0.029**
Number of laborers	0.003	0.031*
cons	−10.477	−0.161
R-squared	0.059	0.118
Hausman	0.016	
Number of obs	4,033	4,033
Number of groups	1,810	1,810

Note: * p < 0.05, ** p < 0.01, *** p < 0.001.

The coefficients (0.240 and 0.275) are significant in both methods for current income growth (Δln*Y*). The coefficients of asset growth (ΔassetIndex) are significant, meaning that wealth contributes to food consumption inequality, similar to previous studies [15,18,21]. In the context of Vietnam’s rapidly changing, an upward trend in assets significantly enhances living standards [6,7]. The results show that food consumption growth is excessively sensitive to income growth and asset growth, consistent with previous studies on Vietnam [1–4].

Household size has a positive effect that means households with a larger number of persons have greater consumption demands. Minority households are particularly apparent in FE column. Nevertheless, the asset index negatively affects consumption in RE models and is not significant in the FE method. Variables such as age, education, year, rural, region and marital status show no impact and do not align with previous studies [16,44,45].

In summary, food consumption growth is explained mainly by income growth, asset accumulation and a few by household size and ethnicity. Some household groups who are vulnerable to these factors need appropriate welfare policies to support food consumption stability in cases of economic fluctuation [53].

### 5.2 Marginal propensity to food consumption growth by asset classification

Equations (5) and (8) are estimated separately to differentiate the role of assets and asset accumulation in the marginal propensity to consume (MPCs). Focusing on MPCs by asset (*A*_*i,t*_) and asset accumulation (*AG*_*i,t*_), demographic factors are simplified, keeping only ethnicity and household size as shown in Table 4. In addition to measuring income changes in the two models, equation (5) examines the impact of the asset index on food consumption growth. While Equation (8) considers the direct effect of growth rate of assets changes on food consumption. The fixed effects (FE) method is used to estimate these equations. Unlike [18], who integrate quintiles within a single model, this estimation calculates MPCs for each quintile, from poor to rich by asset level and from slow to fast by accumulation speed (1–5 groups). With this estimation approach, it is possible to compare the MPCs of poor and wealthy households, as well as slow and fast accumulation rates. The estimation results are described in Tables 5 and 6 below:

**Table 5:** Euler equation by asset index for food consumption growth.

Variable	Quintile 1	Quintile 2	Quintile 3	Quintile 4	Quintile 5
Δln*Y*	0.320***	0.325***	0.216***	0.237***	0.230***
Asset Index	0.007	0.129	0.162*	−0.008	−0.005
Ethnic	−0.072	−0.304	1.039*	−0.375	1.578**
Household size	0.055	0.047	0.094*	0.114***	0.104**
cons	−0.002	0.215	−1.142**	−0.065	−1.759***
R-squared: Overall	0.239	0.095	0.035	0.069	0.018
F test that all *u_i*	0.560	0.660	0.530	0.550	0.560
Number of obs	657	748	835	855	942
Number of groups	360	361	361	362	366

Note: * p < 0.05, ** p < 0.01, *** p < 0.001.

**Table 6:** Euler equation by growth rate of assets for food consumption growth.

Variable	Quintile 1	Quintile 2	Quintile 3	Quintile 4	Quintile 5
Δln*Y*	0.209***	0.288***	0.245***	0.233***	0.229***
ΔAssetIndex	0.056	0.178***	0.117*	0.048	0.172**
Ethnic	1.426**	3.130**	0.511	0.045	−0.356
Household size	0.098***	0.103**	0.027	0.137**	0.018
cons	−1.669***	−3.232***	−0.429	−0.478	0.247
R-squared: Overall	0.066	0.046	0.080	0.073	0.052
F test that all *u_i*	0.530	0.590	0.660	0.490	0.630
Number of obs	877	873	802	750	735
Number of groups	358	359	358	360	375

Note: * p < 0.05, ** p < 0.01, *** p < 0.001.

The result in Table 5 shows that the coefficient has been significant in all quintiles, ranging from 0.216 to 0.325. MPCs are higher for households with fewer assets. Conversely, lower for those with more assets, they are similar in other countries [15,16,18–20]. Notably, the wealthiest households still show high MPCs to explain being vulnerable to economic fluctuations in Vietnamese households [1,3–5]. Clearly, inequality in assets and income has translated into inequality in food consumption.

The asset index has a significant impact in Quintile 3 (0.162), but it is insignificant in other quintiles. Ethnicity and household size are significant effects in certain quintiles. However, factors like asset index, household size, and ethnicity are statistically significant for Quintiles 3, 4, and 5 (from middle to wealthy groups) and insignificant for the poorer quintile. Perhaps in poorer households, a very high MPC plays a crucial role in food consumption decisions, while social factors become more evident in households with higher assets.

The results in Table 6 show that income growth in all quintiles has a statistically significant effect on food consumption growth. The MPCs are slightly different from the rate of asset accumulation for Quintiles 2, 3, and 4. This difference is less pronounced than when classified by asset index in Table 5. Asset growth directly impacts food consumption for households in Quintiles 2, 3, and 5 (moderate and high rate asset accumulation). Ethnicity is significantly with low asset accumulation speeds (Quintiles 1 and 2).

Heterogeneity in MPCs is efficiently classified by quintile of asset index. Vietnamese household food consumption response to income aligns with findings from studies in developed countries [15,19]. Particularly, classification by growth rate of asset, households with moderate accumulation speeds directly affect food consumption with higher MPCs. These households with moderate asset accumulation rates have moved from the cash-on-hand to the middle group [6,7,47,53]. Overview, Tables 5 and 6 show that each asset classification method reveals distinct characteristics. Classification by asset index is the best way to describe heterogeneity in MPCs and inequality of income and food consumption.

### 5.3 Predictable income growth by asset classification

Equations (6) and (9) provide food consumption growth equations based on predicted income shocks. Equations (6) and (9) employ asset index classification and the growth rate of the asset respectively. In Equation (2), first predicted income growth’s value was estimated and then Equations (6) and (9) were estimated. Detailed in Equation (2), the income growth (Δln*Y*) is regressed by lagged income growth (*L*.Δln*Y*) and demographic factors: including rural, year, region, gender of head, ethnicity, age of head, married status, education, household size and number of laborers. The Hausman test selects the Fixed Effects (FE) method indicating an appropriate method. The Equation (2) results indicate that only age head, education, household size, and number of laborers are significant. The regression eliminates noise by keeping only lagged income and the major demographic variables to predict income (Predicted_Δ ln*Y*). Then, equations (6) and (9) are measured for each quintile (1–5 equal poor to rich or low to fast) for two different types of asset classification.

Table 7, the predicted income growth (Predicted_Δln*Y*) is significant in middle-asset households (quintiles 2, 3, and 4), similar to other studies [4,12,18]. For middle-asset households, the predicted income growth explains food consumption growth better. However, the predicted income growth is irrelevant in cash-in-hand and high-wealth households. The middle asset group is directly affected by demographic characteristics as well. Specifically, the asset index exhibits are significant in some quintiles (3, 5). Household size is significant in quintiles 2 and 4. Ethnicity is important in quintile 3. They are proactive and vigilant regarding updates to their income expectations and consumption [4,8,23].

**Table 7:** Predictable income growth by asset index for food consumption growth.

Variable	Quintile 1	Quintile 2	Quintile 3	Quintile 4	Quintile 5
Predicted_Δln*Y*	0.266	0.388***	0.304**	0.212*	0.166
Asset Index	0.015	0.143	0.201*	0.040	0.043**
Ethnic	0.000	−0.305	1.407**	−0.697	1.553
Household size	0.121	0.054	0.073	0.113*	0.144
cons	−0.266	0.198	−1.356**	0.199	−1.989
R-squared: Overall	0.026	0.051	0.016	0.015	0.011
F test that all *u_i*	0.550	0.570	0.550	0.590	0.580
Number of obs	580	671	732	752	818
Number of groups	339	346	352	354	355

Note: * p < 0.05, ** p < 0.01, *** p < 0.001.

The results in Table 8 show that predicted income growth is significant for households with low and medium asset accumulation rates (quintiles 1, 2, and 3), with high coefficients (0.245, 0.393, and 0.200). The growth rate of the asset index (ΔAssetIndex) affects households with medium and high accumulation rates (Quintiles 2, 3, and 5). There is a contrast in food consumption response: households with slow accumulation rates respond through income expectations, while those with fast accumulation rates respond directly to food consumption and are less sensitive to income expectations (perhaps because total income remains low). Demographic factors are significant for households with slow and medium accumulation rates; for instance, ethnicity positively affects Quintiles 1 and 2, and household size is significant in Quintiles 1, 2, and 4.

**Table 8:** Predictable income growth by growth rate of assets for food consumption growth.

Variable	Quintile 1	Quintile 2	Quintile 3	Quintile 4	Quintile 5
Predicted_ΔlnY	0.245**	0.393***	0.200*	0.114	0.139
ΔAssetIndex	0.072	0.215***	0.203***	0.109	0.208**
Ethnic	2.344***	3.287**	−0.493	0.551	−0.448
Household size	0.124***	0.112*	0.020	0.157**	0.021
_cons	−2.655***	−3.395***	0.534	−0.917	0.243
R-squared: Overall	0.044	0.034	0.018	0.010	0.016
F test that all *u*_*i*	0.510	0.034	0.670	0.550	0.600
Number of obs	753	760	706	682	652
Number of groups	340	351	352	354	349

Note: * p < 0.05, *** p < 0.01, *** p < 0.001.

Comparing Tables 7 and 8 shows that changes in expected income by asset index classification and accumulation rate impact only certain groups with higher coefficients. Middle-asset households pay attention to changes in income. Households with a slow asset accumulation rate focus on expected income. The factors of assets and demographics is a strong impact on food consumption, especially slow asset accumulation. Estimates of expectations income shock and current income both reject the Life-Cycle Permanent Income Hypothesis to smoothen food consumption. However, some middle-income households actively update income expectations to adjust food consumption, performing better than low-income households.

### 5.4 Transitory income shocks by asset classification

Equations (7) and (10) estimate transitory income shocks based on classification of asset index and the growth rate of the asset index. Based on the estimates from equation (2), the residuals interpreted as transitory income shocks (*∊*_*i,t*_) were estimated in the previous section to continue with equations (7) and (10). The Fixed Effects (FE) method was chosen to be suitable with the VARHS data. Demographic variables such as age of head, education, household size and number of laborers were also selected from the estimation in equation (2). Likewise, MPCs are estimated for each quintile across different classification levels (low to high). The estimation results are shown in Tables 9 and 10 below.

**Table 9:** Transitory income shocks by asset index for food consumption growth.

Variable	Quintile 1	Quintile 2	Quintile 3	Quintile 4	Quintile 5
Transitory_ shocks	0.371***	0.323***	0.256***	0.250***	0.279***
Asset Index	0.024	0.129	0.183*	0.036	0.039
Ethnic	0.000	−0.259	1.256*	−0.324	1.452*
Household size	0.124	0.121*	0.112*	0.161**	0.165***
_cons	−0.308	−0.090	−1.354*	−0.272	−1.923**
R-squared: Overall	0.101	0.070	0.030	0.061	0.030
F test that all *u_i*	0.530	0.600	0.520	0.580	0.570
Number of obs	580	671	732	752	818
Number of groups	339	346	352	354	355

Note: * p < 0.05, ** p < 0.01, *** p < 0.001.

**Table 10:** Transitory income shocks by growth rate of assets for food consumption growth.

Variable	Quintile 1	Quintile 2	Quintile 3	Quintile 4	Quintile 5
Transitory_ shocks	0.258***	0.318***	0.235***	0.289***	0.278***
ΔAsset Index	0.079	0.216***	0.204***	0.091	0.198**
Ethnic	2.342***	2.905**	−0.405	0.028	−0.281
Household size	0.148***	0.169***	0.058	0.184***	0.059
_cons	−2.706***	−3.229***	0.314	−0.619	0.043
Number of obs	753	760	706	682	652
Number of groups	340	351	352	354	349
R-squared: Overall	0.065	0.053	0.064	0.072	0.058
F test that all *u_i*	0.510	0.610	0.630	0.490	0.620

Note: * p < 0.05, ** p < 0.01, *** p < 0.001.

In Table 9, the coefficients (0.250–0.371) associated with the transitory income shocks are the highest. Heterogeneity in MPCs with poorer households has a higher coefficient than their wealthier counterparts. The transitory income shock coefficient works better than current and predicted income changes, particularly in the MPCs by asset distributions. Unsurprisingly, income shocks are prioritized when measuring food consumption responses in Vietnam [1,3].

Other factors, households in the asset by middle quintile (Quintile 3 with 0.183) are significant. The coefficients of household size are also high across asset groups (Quintile 2, 3, 4 and, 5). The coefficients of ethnicity for Quintiles 3 (1.256) and Quintiles 5 (1.452) are significant. It demonstrates the persistent inequality in income and wealth and household social conditions, which also serve to underpin the inequality in food household consumption [7,47]. For this reason, socio-economic claims are specifically answered by the needs of ethnic minority households [2]. Notably, high MPCs across groups indicate that households still had difficulties in smoothing food consumption, despite better accumulating assets in Vietnam [6,7].

In Table 10, transitory income shock has significant effects on food consumption growth across the quintiles, with high coefficients (0.235 to 0.318). Furthermore, household groups do not reveal clearly how the coefficient MPC differs between a low growth rate versus a fast growth rate. Food consumption growth is better in response to the coefficient of the growth rate of the asset index for moderate and fast asset growth household groups (Quintiles 2, 3, and 5). It is easier to see group by growth rate to know the impact of asset growth on food consumption. Among slower growth rate of asset groups (Quintile 1 and 2), demographic variables and ethnicity show high coefficients (2.342 to 2.905), about ten times the impact of income shocks. Household size has a direct effect on food consumption for Quintiles 1 and 2. However, this clarification of growth rate impresses the role of social factors as an explanatory variable in groups of relatively slow accumulation rates.

Comparing Table 9 with Table 10 reveals different ways affecting food consumption growth across household groups classified by asset index versus growth rate of asset. The key similarity in both tables is that transitory income shocks have a high impact on food consumption growth. In Table 9, MPCs are higher for poorer households, which means that households are classified by asset index, which is a good way and popular in studies. In Table 10, the classification of households by growth rate does not differ effectively from MPCs. However, the classification of households by growth rate does improve the role of other factors—such as asset growth, ethnicity, and household size—in explaining food consumption growth.

### 5.5. Robust check food consumption growth

Essentially, equations (11) and (12) are similar to equation (1), except for the inclusion of the food consumption habit variable (*lag_*Δln*C*). A robustness check is conducted using System-GMM estimation to validate the MPCs because equations initially estimated by the FE method. In addition, Equations (11) and (12) are also emphasizing forecasting ability and explaining food consumption growth. Measuring consumption growth with dynamic data (VARHS) is estimated using System-GMM to address bias caused by endogeneity. Instrumental variables, *z = *ln*Y − *ln*C*, *Dz = z − z*_*t−2*_, lags *L.D*ln*Y* and *L*_*t-4*_*.D*ln*C*, is used. Due to stringent testing and increased lag lengths, the number of surveyed households was reduced to more than 1003 households and 1862 observations. In this estimation model, there are two estimation forms to clarify the dynamics of food consumption growth. Model 1 estimates with the direct inclusion of the asset index. Model 2 estimates the growth rate of the asset. Separating the two estimations helps to reduce the correlation.

A robustness check using System-GMM has resolved endogeneity issues, making the model reliable. Instrumental variables such as Δln*C*, Δln*Y*, ΔAssetIndex, and *z* (ln*Y* − ln*C*) show a high level of autocorrelation. Instrumental variables with larger lags (*L2/5, L3/5*) were applied to estimate System-GMM. Results from the Arellano-Bond tests (sig > 0.5) indicate no significant autocorrelation. Results from the Sargan and Hansen tests (sig > 0.5) support the validity of the instruments used in the System-GMM estimation. Clearly, the System-GMM method improves upon the FE method with the Euler equation for estimating dynamic data, but the FE method for previous estimations is also reliable and valuable. Estimates 1 and 2 have been treated for endogeneity by instrumental variables such as: *z* = ln*Y* - ln*C*, *Dz* = *z – z*_*t-2*_, *L.D*.ln*Y*, and *L*_*t-4*_*.D*.ln*C*, larger lags (*L*2/5, *L*3/5). The correlation between Δln*C* and Δln*Y* does not exceed time *t* = 3 (6 years), but υ_i,t_ has correlation at *t *= 1 and autocorrela*t*ion at *t* = 2. This correlation can be unders*t*ood as the existence of long-term shocks, not just short-term shocks.The estimation by System-GMM has improved the coefficient compared to previous studies in Vietnamese households [1,3,4].

Table 11 shows that Vietnamese household food consumption growth can be measured and explained by changes in income and assets. Food consumption growth is not random, as in the PIH/LCPIH hypothesis, but rather predictable [27–29]. Additionally, it reaffirms that consumption habits, described by the lagged variable for food consumption growth, do not impact current food consumption growth because the coefficient (*L*_*t-2*_.Δln*C*) is insignificant in both estimations 1 and 2, similar to previous micro studies [8,23,31]. The coefficients (estimation 2) in Table 11 for income growth (0.367) and asset growth (0.243) are quite high compared to Table 4. However, it is also noted that Vietnamese households have diversified their food consumption changes by both income and assets, where the role of assets is more significant. Similar to Table 4, the asset index (estimation 1) does not affect food consumption changes, and social factors do not explain food consumption growth.

**Table 11:** Food consumption growth with household data 2008-2018.

Variable	Model 1	Model 2
*L*_*t-2*_. Δln*C*	0.383	1.084
Δln*Y*	0.323***	0.367***
ΔAssetIndex		0.243**
AssetIndex	−0.013	
Age of head	0.000	0.003
Household size	0.068***	0.025
_cons	−0.244	−0.269
Arellano-Bond test for AR(1)	0.065	0.070
Arellano-Bond test for AR(2)	0.791	0.445
Sargan test of overid	0.088	0.065
Hansen test of overid	0.081	0.127
Number of obs	1862	1862
Number of groups	1003	1003

Note: * p < 0.05, ** p < 0.01, *** p < 0.001.

Overview, food consumption growth in Vietnamese households exhibits steady growth rates during an extended time frame (2008–2018). The role of asset accumulation as a means of diversifying responses to change in food consumption instead of only income sources. Although the PIH hypothesis is rejected, the biennial measurement data may lead to a different understanding, such as the concept of permanent income shocks or the distinction between liquid and illiquid assets compared to previous studies.

## 6. Conclusions and policy implications

The study results indicate that Vietnamese household food consumption growth is measured and predicted by income changes and asset growth but not by food consumption habits. Using dynamic data from VARHS (2008–2018), the System-GMM method provides better explanatory indicators than the FE and RE methods, though all methods are reliable. Marginal propensity to food consumption growth (MPCs) is higher for poorer households when they are classified by asset indices. Conversely, demographic factors such as asset accumulation, household size, and ethnicity explain food consumption more significantly when MPCs are classified by growth rates of assets. Additionally, subgroups combined by different classification types will exhibit varying food consumption responses, detailed below.

Heterogeneity in MPCs is quite clear among household groups when they are classified by asset index and break down income changes into categories such as current income, predicted income and transitory income shocks. The MPCs are higher for poorer households than for wealthier ones and high across all groups, especially by transitory income shocks. These mean all household groups are vulnerable, and wealth and income inequality load food consumption inequality.

In addition, classification by growth rate also provides new characteristics for categorizing household food consumption. Especially for the household group with slower growth, the MPC decreases, and other factors become more influential in explaining food consumption. Note that with this method, MPCs show only minor differences across types of income changes, except for predicted income shocks. This phenomenon may be explained by slower growth households tend to be in better conditions, allowing them to diversify buffers for income and food consumption changes. Unlike previous methods that classify MPCs, this classification is first applied in measurement.

Finally, household food consumption is excessively sensitive to income changes, instead of the PIH. The GMM estimation used instrumental variables (lagged at *t* = 3) to deal with endogeneity, yet errors are still persistence shown in the model at *t* = 2.

Future studies should detail error components, which can distinguish the types of shocks (transitory and permanent) with the partial consumption insurance hypothesis. There is a need to investigate households that have trouble amassing assets as a result of specific demographic circumstances. Besides the issues mentioned earlier, liquidity issues and precautionary savings can explain other cause and effect for the next examination.

### Policy implications

The study shows that Vietnam’s food consumption growth trend has improved significantly, along with income growth and asset accumulation. However, low-asset households have reacted to the change in food consumption when faced with shocks. There are some suggestions for policies:

Concentrate on vulnerable groups: the results show that is highest MPC for adverse situations and disadvantaged groups. Therefore, it is necessary to expand and promptly implement cash or food support programs for large size households with few workers, low income, low assets, rural areas and ethnic minorities at economic downturn. Enterprises and NGOs can support through social responsibility activities.

Access to credit and microfinance: the results indicate that poor households respond more to income shocks in their food consumption due to limited resources. Therefore, it is necessary to expand microfinance, flexible savings, and community insurance. The state supports the legal framework and operating costs, while banks and financial institutions develop services suitable for lack of collateral.

Supply product for households: the results shows that households tend to increase their asset and income accumulation, demonstrating the role of asset “buffer” to cope with risks. Purchasing policies help to increase production materials, and durable goods that improve the quality of life and production efficiency. Businesses can access the increasing demand of households not only in urban areas but also in rural areas, including demand related to food consumption.

Optimizing policies according to group differences: The household heterogeneity, such as poor and well-off households, households with fast and slow asset accumulation, and regions, shows in the results. These characteristics should be added to the index to classify and assess the economic status of households. Design policies based on the indicators of income and asset trends to quickly identify household needs and support food consumption stability.

## Supporting information

S1 File Code. (DO)

S2 File Data. (CSV)

S3 File Readme. (TXT)
